# MicroRNA-488 and -920 regulate the production of proinflammatory cytokines in acute gouty arthritis

**DOI:** 10.1186/s13075-017-1418-6

**Published:** 2017-09-15

**Authors:** Weidong Zhou, Ying Wang, Rongfeng Wu, Yan He, Qun Su, Guixiu Shi

**Affiliations:** The First Affiliated Hospital of Xiamen University, No. 55 Zhenhai Road, Xiamen, 361003 China

**Keywords:** MicroRNA, Gouty arthritis, Proinflammatory cytokines, Interleukin-1β, Monosodium urate crystals

## Abstract

**Background:**

Gout is considered one of the most painful acute conditions caused by deposition of monosodium urate (MSU) crystals within joints. Recent studies have shown that interleukin (IL)-1β is a key inflammatory mediator in acute gouty arthritis (GA), and its level is regulated by microRNAs (miRNAs). However, the molecular mechanisms of the regulation remain unclear.

**Methods:**

A miRNA microarray was used to analyze the miRNA expression profiles in peripheral white blood cells (WBCs) of patients with GA. THP-1 cells were transfected with miRNA mimics, stimulated by MSU crystals, and then subjected to quantitative real-time polymerase chain reaction or Western blot analysis. Levels of IL-1β, IL-8, and tumor necrosis factor (TNF)-α in culture supernatants of THP-1 cells were measured by enzyme-linked immunosorbent assay. A luciferase reporter assay was conducted to confirm the interaction of miRNA and IL-1β 3′-untranslated regions (UTRs).

**Results:**

Combining bioinformatics and miRNA expression profiles, we found five miRNAs (hsa-miR-30c-1-3p, hsa-miR-488-3p, hsa-miR-550a-3p, hsa-miR-663a, and hsa-miR-920) that possibly target IL-1β. Then, we demonstrated that miR-488 and miR-920 were significantly decreased in the WBCs of patients with GA and that MSU crystals could inhibit expression of miR-488 and miR-920. Upregulation of miR-488 and miR-920 could suppress MSU-induced IL-1β protein expression in THP-1 cells, but no significant difference in IL-1β messenger RNA levels was observed. Moreover, we found that miR-488 and miR-920 could directly target the 3′-UTR of IL-1β. Overexpression of miR-488 and miR-920 could significantly inhibit the gene and protein expression of IL-8 and TNF-α in MSU-induced THP-1 cells.

**Conclusions:**

This study demonstrates the roles of miR-488 and miR-920 in regulating the production of proinflammatory cytokines in the pathogenesis of GA. These findings suggest that miR-488 and miR-920 could serve as potential therapeutic targets in the treatment of GA.

**Electronic supplementary material:**

The online version of this article (doi:10.1186/s13075-017-1418-6) contains supplementary material, which is available to authorized users.

## Background

Gout is considered one of the most painful acute conditions and is caused by deposition of monosodium urate (MSU) crystals within joints and periarticular soft tissues as a result of hyperuricemia [1]. MSU crystals released from preformed deposits in the joints can be phagocytosed by monocytic inflammatory cells. This triggers the release of proinflammatory cytokines such as interleukin (IL)-1β, tumor necrosis factor (TNF)-α, IL-6, IL-8, CCL2, and interferon-γ [2, 3]. Recent studies have suggested that IL-1β is a crucial inflammatory mediator induced by MSU crystals, indicating that IL-1β may serve as a potential therapeutic target for the treatment of acute gouty arthritis (GA) [4, 5].

MicroRNAs (miRNAs, miRs) are evolutionarily conserved small noncoding RNA molecules that function as negative post-transcriptional gene regulators [6]. Because of the ability of a single miRNA molecule to target hundreds of messenger RNAs (mRNAs), abnormal miRNA expression is associated with the initiation of many diseases [7]. Recent studies have suggested that miRNAs may be involved in the development of GA [8, 9]. However, the molecular mechanisms of miRNAs in GA are still unclear. Given the important roles of miRNAs in inflammatory diseases, including GA, more studies on their roles are needed [10].

The aim of this study was to find any miRNAs participating in regulating the pathogenesis of GA. In the present study, a miRNA microarray and bioinformatics were used to analyze the miRNA expression profiles in peripheral white blood cells (WBCs) of patients with GA. We found that miR-488 and miR-920 were significantly decreased in the WBCs of patients with GA and that MSU crystals could inhibit expression of miR-488 and miR-920. We further demonstrated that miR-488 and miR-920 could directly target the 3′-untranslated region (3′-UTR) of IL-1β. Therefore, we proposed a regulatory mechanism of miR-488 and miR-920 in proinflammatory cytokine production in GA.

## Methods

### Patients and samples

The diagnosis of GA was made according to the 1977 American College of Rheumatology classification criteria [11]. Samples of peripheral blood were obtained from patients with GA (*n* = 10) and healthy control subjects (HCs; *n* = 10). The clinical data and specifics of these patients are summarized in Table 1. The presence of MSU crystals in all synovial fluid samples of patients with GA was confirmed by polarizing light microscopy.

**Table 1:** Clinical features of patients with acute gouty arthritis

Parameters	Healthy control subjects (*n* = 10)	Patients with GA (*n* = 10)
Male, *n* (%)^a^	10 (100%)	10 (100%)
Age, years, mean (range)^a^	40 (27 to 60)	48 (30 to 66)
Duration of acute gout, days, mean (range)	N/A	3 (2 to 5)
Comorbidity	N/A	
Hypertension, *n* (%)		3 (30%)
Hyperglycemia, *n* (%)		2 (20%)
Hypercholesterolemia, *n* (%)		4 (40%)
Current medicines	N/A	
Naive, *n* (%)		10 (100%)
Serum urate level, μmol/L, mean (range)	404 (265 to 490)	559 (444 to 696)
ESR, mm/h, mean (range)	N/A	62 (46 to73)
CRP, mg/dl, mean (range)	N/A	18.4 (5.9 to 71)

*CRP* C-reactive protein, *ESR* Erythrocyte sedimentation rate, *N/A* Not applicable

^a^Not significant

Peripheral venous blood samples were collected in heparin-containing tubes. WBCs were isolated by using red blood cell lysis buffer (Solarbio, Beijing, China). The collected WBCs (~1 × 10^7^ cells) were then lysed by adding 1 ml of TRIzol Reagent (Life Technologies, Carlsbad, CA, USA) for the exaction of total RNA. This study was approved by the Ethics Committee of the First Affiliated Hospital of Xiamen University. All patients provided written informed consent.

### Cell culture, treatment, and transfection

The human monocytic THP-1 cell line was cultured in Gibco RPMI 1640 medium (Life Technologies) supplemented with 10% Gibco FBS, 100 IU/ml penicillin, and 100 μg/ml streptomycin in a humidified 5% CO_2_ atmosphere at 37 °C. THP-1 cells were plated at the density of 1.5 × 10^6^/ml in six-well plates. The THP-1 cells were stimulated for 3 h with 0.5 μM phorbol 12-myristate 13-acetate (PMA; Sigma-Aldrich, St. Louis, MO, USA) the day before stimulation. This treatment increases the phagocytic properties of the cells and induces a constitutive production of proinflammatory cytokines [4]. Then, the cells were stimulated with different concentrations of MSU crystals (InvivoGen, San Diego, CA, USA) for 24 h, the culture supernatants were collected for proinflammatory cytokine detection, and the cells were washed once in RPMI medium for RNA isolation or protein extraction.

To overexpress miRNAs (hsa-miR-30c-1-3p, hsa-miR-488-3p, hsa-miR-550a-3p, hsa-miR-663a, and hsa-miR-920), the corresponding miRNA mimics or negative control (NC) mimics (50 nM) were transfected into THP-1 cells using Lipofectamine RNAiMAX Transfection Reagent (Life Technologies) in accordance with the manufacturer’s instructions. After 24 h of transfection, cells were stimulated for 3 h with 0.5 μM PMA. Then, cells were washed and stimulated with 250 μg/ml MSU crystals for 24 h to detect the production of proinflammatory cytokines. miRNA mimics and their NC mimics were synthesized by RiboBio (Guangzhou, China).

### RNA extraction, reverse transcription, and quantitative real-time polymerase chain reaction

RNA extraction, reverse transcription, and quantitative real-time polymerase chain reaction (qRT-PCR) were performed as previously described [12]. Briefly, total RNA was extracted using TRIzol reagent according to the manufacturer’s instructions (Life Technologies). For mature miRNA expression analysis, Bulge-Loop™ miRNA qRT-PCR primer sets (one reverse transcription primer and a pair of qPCR primers for each set) specific for corresponding miRNAs (hsa-miR-30c-1-3p, hsa-miR-488-3p, hsa-miR-550a-3p, hsa-miR-663a, and hsa-miR-920) and RNU6B (U6) were designed by and purchased from RiboBio. The miRNA bulge-loop was reverse transcribed using the PrimeScript RT Reagent Kit (TaKaRa, Dalian, China) and quantified by qPCR using the SYBR Premix Ex Taq (TaKaRa). U6 was used as the internal control. The mRNA quantification was similar to the miRNA quantification, except that complementary DNA was reverse transcribed using oligo(dT)_18_ primer. All qRT-PCR was performed using an ABI 7500 system (Applied Biosystems, Foster City, CA, USA). PCR parameters were as follows: 95 °C for 3 minutes, followed by 40 cycles of 95 °C for 10 seconds, 60 °C for 20 seconds, and 72 °C for 1 second. Melting curve analysis was performed at the end of the PCR cycles. The primers used were as follows: IL-1β sense: 5′-ACGATGCACCTGTACGATCA-3′; IL-1β antisense: 5′-TCTTTCAACACGCAGGACAG-3′; IL-8 sense: 5′-GCATAAAGACATACTCCAAACC-3′; IL-8 antisense: 5′-ACTTCTCCACAACCCTCTG-3′; TNF-α sense: 5′-CAGAGGGAAGAGTTCCCCAG-3′; TNF-α antisense: 5′-CCTTGGTCTGGTAGGAGACG-3′; glyceraldehyde 3-phosphate dehydrogenase (GAPDH) sense: 5′-GGAAGGTGAAGGTCGGAGTCA-3′; GAPDH antisense: 5′-GTCATTGATGGCAACAATATCCACT-3′. The specific gene expression was calculated by using the 2^−ΔΔCT^ method with GAPDH as the calibrator [13].

### miRNA microarray

We analyzed the miRNA expression profiles in WBCs of patients with GA (*n* = 3) and HCs (*n* = 3) by using a miRNA microarray, the miRCURY™ LNA Array (version 18.0) according to the manufacturer’s instructions (Exiqon, Vedbaek, Denmark). Briefly, the RNA was labeled using the miRCURY™ Hy3™/Hy5™ Power labeling kit and hybridized on the miRCURY™ LNA Array. The slides were scanned using the GenePix 4000B microarray scanner (Molecular Devices, Sunnyvale, CA, USA) following the washing steps. Scanned images were then imported into GenePix Pro 6.0 software files (Molecular Devices) for grid alignment and data extraction. Replicated miRNAs were averaged, and miRNAs with intensities ≥ 30 in all samples were chosen for calculating the normalization factor. Expressed data were normalized using median normalization. After normalization, significant differentially expressed miRNAs were identified by volcano plot filtering. Finally, hierarchical clustering was performed to show distinguishable miRNA expression profiling among samples.

### Enzyme-linked immunosorbent assay

Concentrations of IL-1β, IL-8, and TNF-α protein in culture supernatants of THP-1 cells were measured using commercially specific enzyme-linked immunosorbent assay (ELISA) kits following the manufacturer’s instructions (NeoBioscience Technology Co., Beijing, China). The sensitivities of the assays were 3 pg/ml, and the intraassay and interassay coefficients of variation were < 8%. Each sample was assayed in duplicate.

### Western blot analysis

Total protein from WBCs and cultured cells was extracted using radioimmunoprecipitation assay buffer (Thermo Fisher Scientific, Rockford, IL, USA). Then, protein concentration was determined by using a bicinchoninic acid assay (Thermo Fisher Scientific). Whole-cell lysates were separated by 10% sodium dodecyl sulfate-PAGE, and the proteins were transferred to polyvinylidene difluoride membranes by electroblotting. Nonspecific binding was blocked by incubating the membranes in 5% nonfat milk in Tris-buffered saline with Tween-20 (50 mmol/L Tris-HCl, 150 mmol/L NaCl, 0.1% Tween-20) for 1 h at room temperature. Membranes were incubated overnight at 4 °C with IL-1β antibody (OriGene Technologies, Rockville, MD, USA) and β-actin antibody (BBI Solutions, Cardiff, UK), both at a 1:1000 dilution. The membranes were subsequently incubated with a horseradish peroxidase-conjugated secondary antibody (1:10000; Thermo Fisher Scientific) for 1 h at room temperature and visualized using enhanced chemiluminescence (Thermo Fisher Scientific) and x-ray film.

### Luciferase reporter assay

The putative binding sites of miRNAs in the 3′-UTR of the human IL-1β gene transcript were predicted by combinatorial use of three different algorithms, including TargetScan (http://www.targetscan.org/), miRanda (http://www.microrna.org/), and PicTar (http://pictar.mdc-berlin.de/). Direct targeting of the IL-1β 3′-UTR was determined by cloning of the 3′-UTR seed regions and mutated seed regions into separate pGL3 luciferase reporter vectors (Promega, Madison, WI, USA). The primers selected are shown in Additional file 1: Table S1. Dual-luciferase reporter assays (Promega) were performed according to the manufacturer’s instructions. Briefly, HEK 293 T cells were seeded into 24-well plates in triplicate and cultured for 24 h. The cells were cotransfected with indicated plasmids and 1 ng of pRL-TK Renilla by using Lipofectamine 2000 Transfection Reagent, and firefly luciferase activities were normalized to Renilla luciferase activities. miRNA function was expressed as a percent reduction in the luciferase activity of cells transfected with the reporter vector containing the corresponding miRNA target sequences compared with cells transfected with the vector without the corresponding miRNA target. Experiments were repeated three times in triplicate.

### Statistical analysis

All statistical analyses were performed using SPSS software (version 16.0; SPSS, Chicago, IL, USA), and graphics were generated using Prism software (version 5.0; GraphPad Software, La Jolla, CA, USA). Multiple group comparisons were analyzed by one-way analysis of variance. Differences between two groups were analyzed by Student’s *t* test. All experiments were repeated in triplicate with independent assays. All data are shown as mean ± SEM. *P* < 0.05 was considered statistically significant.

## Results

### Findings and analysis of candidate miRNAs in regulating IL-1β expression in human gouty arthritis

**Fig. 1:**
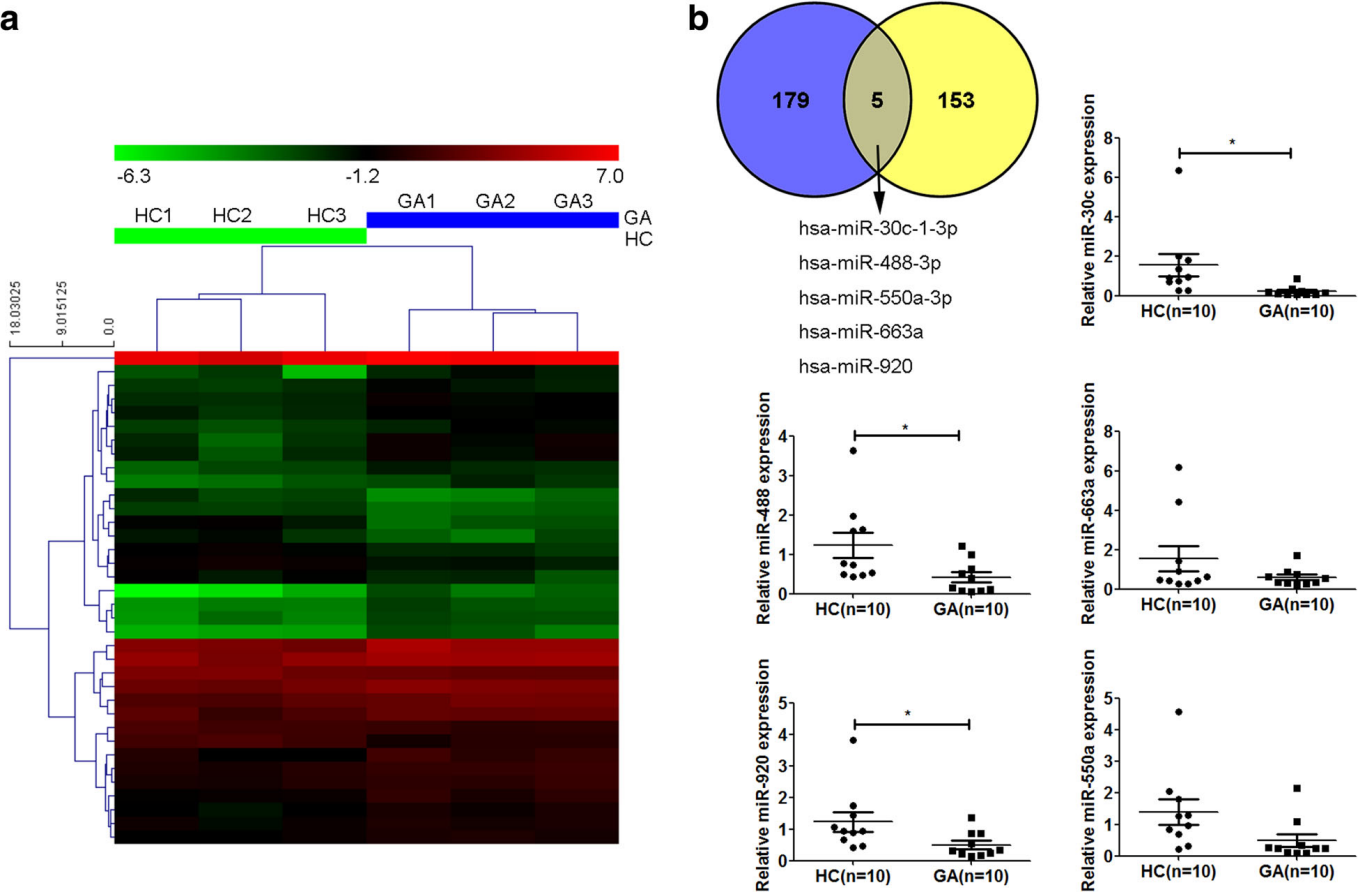
Identification of microRNAs (miRNAs, miRs) regulating interleukin (IL)-1β expression in human gouty arthritis. **a** Heat map displays the most differential expression levels in the healthy control subjects (HCs; *n* = 3) and patients with acute gouty arthritis (GA; *n* = 3). The color key indicates increasing miRNA expression levels from *red* to *green* compared with HC. **b** Venn diagram (*upper left*) shows five miRNAs possibly targeting IL-1β in the peripheral white blood cells (WBCs) of patients with GA. *Blue* represents 184 potential miRNAs targeting human IL-1β gene as predicted by bioinformatics. *Yellow* represents microarray results of 158 miRNAs markedly downregulated in the WBCs of patients with GA. The expression levels of the five selected miRNAs were detected by quantitative real-time polymerase chain reaction in HCs (*n* = 10) and patients with GA (*n* = 10). Values are expressed as mean ± SEM of three independent experiments, each of which was run in triplicate. *P < 0.05

IL-1β is a key proinflammatory cytokine in gouty inflammation. In our previous work, we found that IL-1β was significantly upregulated in the WBCs of patients with GA (*see* Additional file 2). In the present study, we analyzed the miRNA expression profiles in WBCs of patients with GA (*n* = 3) and HC (*n* = 3) by using a miRNA microarray (Fig. 1a). According to the mechanism of negative regulation between miRNA and its target gene, we screened 158 miRNAs that were markedly downregulated in the WBCs of patients with GA.

Furthermore, to search for potential miRNAs that directly target human IL-1β, we used a bioinformatic prediction approach for putative binding sites of miRNAs in the 3′-UTR of the human IL-1β gene. Several prediction algorithms, such as miRWalk (http://zmf.umm.uni-heidelberg.de/apps/zmf/mirwalk2/), TargetScan (http://www.targetscan.org), miRDB (http://www.mirbase.org/), and miRanda (http://www.microrna.org/), were used for this purpose. As a result, 184 potential miRNAs targeting the human IL-1β gene were found. Combining analysis of bioinformatic prediction and miRNA array results, we found five miRNAs (hsa-miR-30c-1-3p, hsa-miR-488-3p, hsa-miR-550a-3p, hsa-miR-663a, and hsa-miR-920) possibly targeting IL-1β that were decreased in the WBCs of patients with GA (Fig. 1b). Thus, we further used qRT-PCR to test the expression levels of these five miRNAs. The results showed that the expression levels of hsa-miR-30c-1-3p, hsa-miR-488-3p, and hsa-miR-920 were significantly decreased in patients with GA (*n* = 10) compared with that of HC (*n* = 10) (*P* < 0.05) (Fig. 1b).

### Effects of MSU crystals on expression of miRNAs and proinflammatory cytokines in monocytic THP-1 cells

**Fig. 2:**
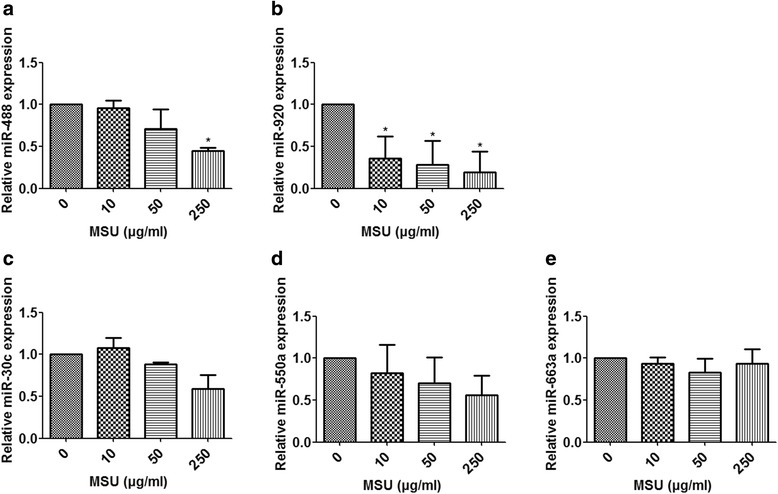
Effects of monosodium urate (MSU) crystals on expression of microRNAs (miRNAs, miR) in monocytic THP-1 cells. THP-1 cells were stimulated by the indicated concentration of MSU crystals. The expression levels of miRNAs were detected by quantitative real-time polymerase chain reaction﻿(**a**-**e**)﻿. Values are expressed as mean ± SEM of three independent experiments, each of which was run in triplicate. *P < 0.05 versus no MSU stimulation

To further confirm the effects of MSU on expression of the five selected miRNA, MSU was used to stimulate the monocytic THP-1 cells in vitro. The results showed that MSU (250 μg/ml) significantly inhibited expression of miR-488 and miR-920 (*P* < 0.05) (Fig. 2a and b). However, the MSU (250 μg/ml) did not significantly regulate expression of miR-30c-1, miR-550a, or miR-663a (*P* > 0.05) (Fig. 2c–e).

**Fig. 3:**
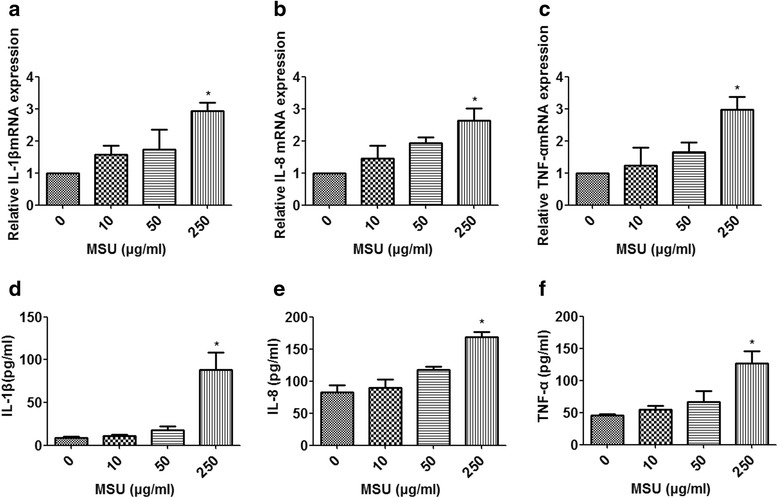
Monosodium urate (MSU) crystals promote the expression of proinflammatory cytokines in THP-1 cells. THP-1 cells were stimulated by the indicated concentration of MSU crystals. The messenger (mRNA) expression of interleukin (IL)-1β, IL-8, and tumor necrosis factor (TNF)-α were detected by quantitative real-time polymerase chain reaction (**a**–**c**). Protein expression of IL-1β, IL-8, and TNF-α was detected by enzyme-linked immunosorbent assay (**d**–**f**). Values are expressed as mean ± SEM of three independent experiments, each of which was run in triplicate. *P < 0.05 versus no MSU stimulation

In addition, we analyzed the effects of MSU on expression of proinflammatory cytokines, such as IL-1β, IL-8, and TNF-α. qRT-PCR analysis showed that MSU (250 μg/ml) could induce mRNA expression of IL-1β, IL-8, and TNF-α (*P* < 0.05) (Fig. 3a–c). Similarly, the ELISA results showed that MSU (250 μg/ml) could also increase the secretion of IL-1β, IL-8, and TNF-α (*P* < 0.05) (Fig. 3d–f).

### miR-488 and miR-920 suppress MSU-induced IL-1β protein expression in monocytic THP-1 cells

**Fig. 4:**
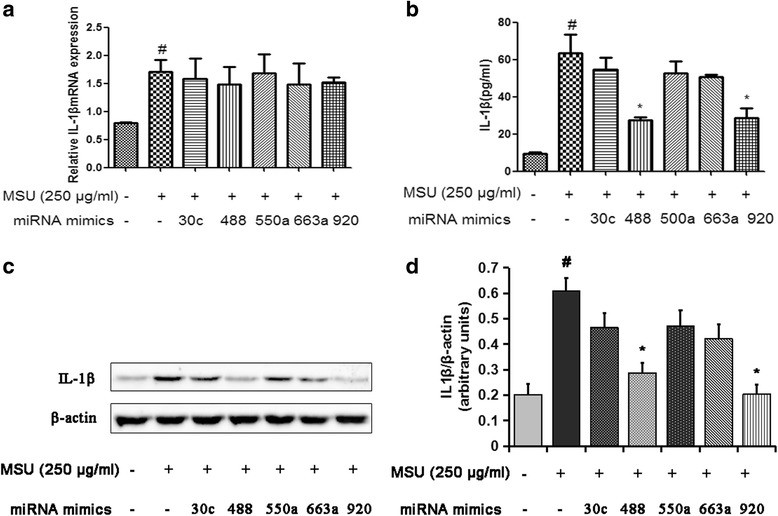
MicroRNA (miR, miRNA)-488 and miR-920 inhibit monosodium urate (MSU)-induced interleukin (IL)-1β protein expression in monocytic THP-1 cells. The miRNA mimics or negative control (NC) mimics (50 nM) were transfected into THP-1 cells using Lipofectamine RNAiMAX reagent in accordance with the manufacturer’s instructions. After 24 h of transfection, cells were stimulated for 3 h with 0.5 μM 12-myristate 13-acetate. Then, cells were washed and stimulated with 250 μg/ml MSU crystals for 24 h to detect the production of IL-1β. After the treatment, the cells were collected and analyzed by quantitative real-time polymerase chain reaction (**a**) or Western blotting (**c**). The cell culture supernatants were collected to detect the concentrations of IL-1β by enzyme-linked immunosorbent assay (**b**). **d** Densitometric analysis of immunoblot band intensities for IL-1β normalized by β-actin. Values are expressed as mean ± SEM of three independent experiments. ^#^ *P* < 0.05 versus NC, *P < 0.05 versus MSU stimulation alone

To further evaluate the effects of the five selected miRNAs (hsa-miR-30c-1-3p, hsa-miR-488-3p, hsa-miR-550a-3p, hsa-miR-663a, and hsa-miR-920) on IL-1β expression, transfection experiments were performed. We transfected the THP-1 cells with miRNA mimics or NC. Compared with the NC, transfection with 50 nM of the five selected miRNA mimics in THP-1 cells led to dramatic increases in the corresponding miRNA expression as detected by qRT-PCR (*see* Additional file 3). We further found that there was no significant difference in IL-1β mRNA level after transfecting with corresponding miRNA mimics (Fig. 4a). However, both miR-488 and miR-920 could significantly inhibit MSU-induced IL-1β protein level in monocytic THP-1 cells as detected by ELISA and Western blotting (*P* < 0.05) (Fig. 4b–d). These results suggest that miR-488 and miR-920 can suppress IL-1β expression at the posttranscriptional level.

### miR-488 and miR-920 bind directly to the 3′-UTR of IL-1β

**Fig. 5:**
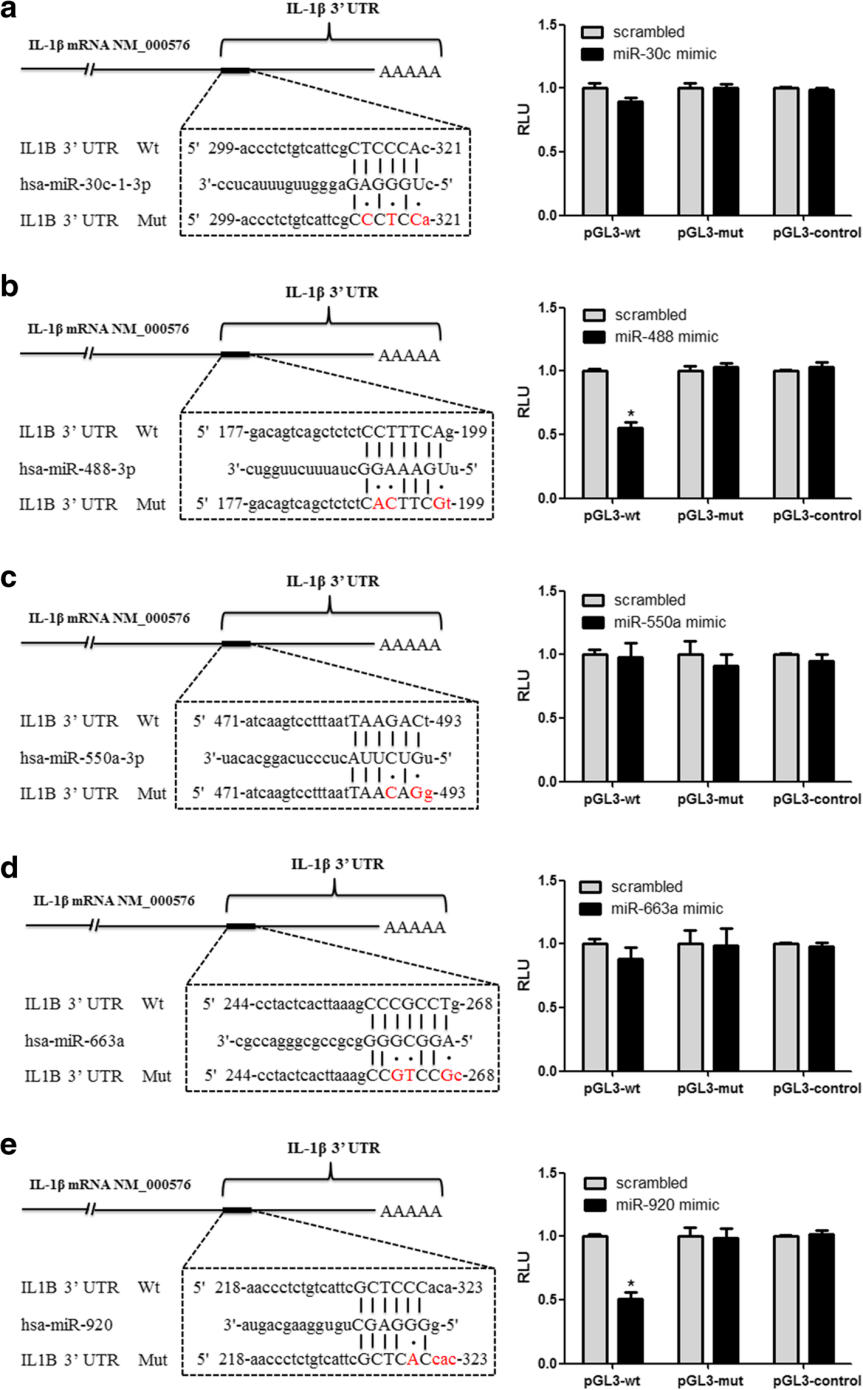
MicroRNA (miR, miRNA)-488 and miR-920 directly target the 3′-untranslated region (3′-UTR) of interleukin (IL)-1β. Sequence alignments of the miRNA base pair site in the 3′-UTR of IL-1β messenger RNA (mRNA) are shown in the *left figures*﻿ (**a**-**e**)﻿. The “seed” sequence of miRNA that is complementary to IL-1β is shown in capital letters in the *dashed boxes*. The mutant sequence (mut) is identical to the wild-type sequence (wt), except the mutated nucleotides are shown in *red*. Results of luciferase assays with HEK 293 T cells are shown in the *right figures* (﻿**a﻿**-﻿**e﻿**). Cells were cotransfected with wt/mut 3′-UTR with miRNA mimics or negative control as indicated. Twenty-four hours after transfection, luciferase activity was detected using a dual-luciferase reporter assay system according to the manufacturer’s instructions. Values are expressed as mean ± SEM of three independent experiments, each of which was run in triplicate. *P < 0.05 versus scrambled group. *RLU* Relative light units

To determine whether the selected miRNAs inhibit IL-1β expression by directly binding with the IL-1β 3′-UTR, we constructed luciferase reporter plasmid vectors of the IL-1β 3′-UTR containing the predicted binding sites (wild type [wt]) or mutated sequences (mutant type [mut]) of selected miRNAs. Then, the plasmids and the miRNA mimics were cotransfected into HEK 293 T cells, and dual-luciferase reporter assays were used to detect changes in luciferase activity. The results showed that cotransfection of miR-488 or miR-920 mimics and the wt plasmid significantly decreased luciferase activity but not that of the mut reporter, indicating that miR-488 and miR-920 can bind directly to the 3′-UTR of IL-1β (Fig. 5b, e). However, cotransfection of the other three miRNAs mimics (hsa-miR-30c-1-3p, hsa-miR-550a-3p, and hsa-miR-663a) and their wt plasmid did not significantly change luciferase activity (Fig. 5a, c, d).

### miR-488 and miR-920 inhibit expression of proinflammatory cytokines in MSU-induced monocytic THP-1 cells

**Fig. 6:**
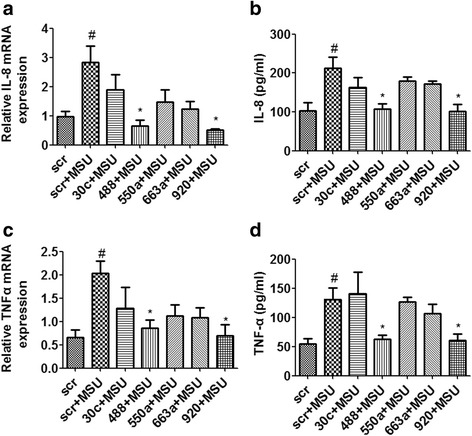
MicroRNA (miR, miRNA)-488 and miR-920 suppress monosodium urate (MSU)-induced expression of proinflammatory cytokines in THP-1 cells. The miRNA mimics or negative control (NC) mimics (50 nM) were transfected into THP-1 cells using Lipofectamine RNAiMAX reagent in accordance with the manufacturer’s instructions. After 24 h of transfection, cells were stimulated for 3 h with 0.5 μM 12-myristate 13-acetate. Then, cells were washed and stimulated with 250 μg/ml MSU crystals for 24 h to detect the production of proinflammatory cytokines. After the treatment, the cells were collected and analyzed by quantitative real-time polymerase chain reaction (**a**, **c**). The cell culture supernatants were also collected to detect the concentrations of interleukin (IL)-8 and tumor necrosis factor (TNF)-α by enzyme-linked immunosorbent assay (**b**, **d**). Values are expressed as mean ± SEM of three independent experiments, each of which was run in triplicate. ^#^ *P* < 0.05 versus scrambled (scr) group, *P < 0.05 versus MSU stimulation alone. *mRNA* Messenger RNA

To further investigate the effects of selected miRNAs on expression of proinflammatory cytokines, we transfected the miRNA mimics into MSU-induced THP-1 cells and detected the mRNA and protein expression of IL-8 and TNF-α by qRT-PCR and ELISA, respectively. The results showed that overexpression of miR-488 and miR-920 could significantly inhibit the gene and protein expression of IL-8 and TNF-α in MSU-induced THP-1 cells in comparison with that of NC transfected cells (*P* < 0.05) (Fig. 6a–d). However, compared with NC, transfection with the other three miRNAs mimics (hsa-miR-30c-1-3p, hsa-miR-550a-3p, and hsa-miR-663a) in MSU-induced THP-1 cells did not lead to a significant difference in either gene or protein expression of IL-8 and TNF-α (Fig. 6a–d).

## Discussion

Gout is the most common inflammatory arthritic disease. It is caused by precipitation of MSU crystals in the joint and is associated with impaired quality of life [14]. Although MSU was first identified in gout hundreds of years ago, the mechanisms by which MSU crystals trigger acute inflammation have only recently begun to be understood and still are unclear. MSU crystals can induce a variety of cytokines, including IL-1β, IL-6, IL-8, and TNF-α [15]. Among these cytokines, IL-1β is suggested to play a key role in GA [16]. Clinical studies have shown that selective blockade of IL-1β effectively inhibits pain and inflammation in patients with GA refractory to other treatments [17–19]. These studies suggest that IL-1β may be a potential therapeutic target in GA. Recent studies have revealed that MSU crystal-induced IL-1β production is initiated by Toll-like receptor pathways and the NLRP3 inflammasome [4, 20]; however, there are no reports of miRNAs directly targeting IL-1β.

miRNAs are defined as endogenous small noncoding RNAs that play a crucial regulatory role by binding to the mRNAs of protein-coding genes to mediate posttranscriptional repression [21, 22]. Thousands of miRNAs have been identified, and emerging data suggest that miRNAs have important roles in a wide range of human diseases [23]. miRNAs have been shown to play essential regulatory roles in the innate immune system [24]. Recently, studies confirmed that miRNAs could regulate IL-1β expression in several ways, thereby affecting inflammatory responses of the body [25–27]. Also, recent research indicates that miRNAs are involved in the development of inflammatory arthritis, including GA [8, 9, 28]. However, according to current literature, no miRNAs have been identified as directly targeting IL-1β. In the present study, by combining analysis of bioinformatics prediction and miRNA expression profiles in WBCs of GA, we found five miRNAs (hsa-miR-30c-1-3p, hsa-miR-488-3p, hsa-miR-550a-3p, hsa-miR-663a, and hsa-miR-920) that possibly target IL-1β. Moreover, we demonstrated that miR-488 and miR-920 were significantly decreased in the WBCs of patients with GA and that MSU crystals could inhibit expression of miR-488 and miR-920, indicating that reduced expression of miR-488 and miR-920 is involved in the development of GA.

In this study, we found that MSU could induce the mRNA and protein expression of proinflammatory cytokines, such as IL-1β, IL-8, and TNF-α. Recent studies also showed that the exposure of monocyte cell lines to MSU crystals led to the production of proinflammatory cytokines, in particular IL-1β [29, 30]. Phagocytosis of MSU crystals by the monocytes/macrophages plays an important role in this process [4]. It is suggested that MSU-induced inflammatory response is specifically initiated by NLRP3 inflammasome activation, which results in the production of active IL-1β. Active IL-1β is released into the extracellular joint fluid of patients with gout. Then, IL-1β activates IL-1 receptors on endothelial cells and resident macrophages within the joint, mediated by the nuclear factor kappa light chain enhancer of activated B cells signaling pathway and leading to the production of proinflammatory cytokines [20]. A recent study demonstrated that MSU crystals alone were unable to induce IL-1β release from peripheral blood mononuclear cells; however, in the presence of free fatty acids and MSU, large amounts of active IL-1β were detected [31].

Previous studies have shown that miRNA-155 is a proinflammatory regulator through SHIP-1 downregulation in GA and that overexpression of miR-155 can lead to inhibit SHIP-1 levels and increase proinflammatory cytokine expression, including IL-1β [8]. In addition, Dalbeth et al. [9] found that overexpression of miR-146a inhibited MSU-induced IL-1β, TNF-α, monocyte chemoattractant protein-1, and IL-8 gene expression in THP-1 cells, suggesting that miR-146a is a transcriptional brake that is lost during the acute inflammatory response to MSU crystals. Moreover, there are also several miRNAs that can indirectly suppress the production of IL-1β, such as miR-223 and miRNA-146b-5p [32, 33]. In the present study, we found that upregulation of miR-488 and miR-920 could suppress MSU-induced IL-1β protein expression in THP-1 cells, but there was no significant difference in IL-1β mRNA level, suggesting a posttranscriptional regulatory role of miR-488 and miR-920 in GA.

IL-1β is a cytokine encoded by the *IL1B* gene. miRNAs can pair with partially complementary sites in the 3′-UTRs of target mRNAs and lead to translational repression [34]. To address the molecular mechanisms involved in the miRNA-mediated regulation of IL-1β expression, five miRNAs (hsa-miR-30c-1-3p, hsa-miR-488-3p, hsa-miR-550a-3p, hsa-miR-663a, and hsa-miR-920) were predicted to target the 3′-UTR of the *IL1B* gene by miRNA microarray and bioinformatic analysis. Furthermore, the luciferase activity assay showed that among the five miRNAs, miR-488 and miR-920 could directly target the 3′-UTR of the *IL1B* gene and thereby suppressed the expression of IL-1β. In addition, we found that upregulation of miR-488 and miR-920 could significantly inhibit the gene and protein expression of IL-8 and TNF-α in MSU-induced THP-1 cells, suggesting that miR-488 and miR-920 regulate the proinflammatory responses in GA by targeting the *IL1B* gene.

Our findings suggest that miR-488 and miR-920 could serve as potential therapeutic targets for the treatment of GA. However, a limitation of our study is that we did not investigate the effect of miR-488 and miR-920 in an animal model of GA. Future studies should be focused on whether upregulating miR-488 and miR-920 could improve the development of GA in vivo.

## Conclusions

Combining bioinformatic and miRNA array analysis, we found that miR-488 and miR-920 were significantly decreased in GA. Moreover, our findings reveal that miR-488 and miR-920 may suppress the production of proinflammatory cytokines in GA by targeting the 3′-UTR of IL-1β. On the basis of these results, we propose that miR-488 and miR-920 may act as regulators in the development of GA and serve as novel therapeutic targets for treatment.

## Additional files

Additional file 1: Table S1. Primer sequences used for the amplification of IL-1β 3′UTR. (DOCX 15 kb)

Additional file 2: IL-1β gene expression in the peripheral white blood cells of patients with acute gouty arthritis (GA, *n* = 14) and of healthy control subjects (HC, *n* = 10). IL-1β mRNA expression was detected by quantitative real-time PCR. Each bar shows the mean ± SEM. (DOCX 48 kb)

Additional file 3: THP-1 cells were transfected with 50 nM of miRNA mimics or negative control (scrambled) by using Lipofectamine RNAiMAX reagent. Expression of miRNAs was detected by quantitative real-time PCR. Each bar shows the mean ± SEM. (DOCX 31 kb)

## Abbreviations

CRP: C-reactive protein
ELISA: Enzyme-linked immunosorbent assay
ESR: Erythrocyte sedimentation rate
GA: Gouty arthritis
GAPDH: Glyceraldehyde 3-phosphate dehydrogenase
HC: Healthy control
IL: Interleukin
miRNA/miR: MicroRNA
mRNA: Messenger RNA
MSU: Monosodium urate
mut: Mutant type
N/A: Not applicable
NC: Negative control
PMA: Phorbol 12-myristate 13-acetate
qRT-PCR: Quantitative real-time polymerase chain reaction
RLU: Relative light units
TNF: Tumor necrosis factor
UTR: Untranslated region
WBC: Peripheral white blood cell
Wt: Wild type
