# Supplementary material for: MicroRNA-488 and -920 regulate the production of proinflammatory cytokines in acute gouty arthritis
Source: Arthritis Res Ther. 2017 Sep 15;19:203. doi: 10.1186/s13075-017-1418-6 (PMC5602958; doi:10.1186/s13075-017-1418-6)
Supplement: Supplementary file 1 — Primer sequences used for the amplification of IL-1β 3′UTR. (DOCX 15 kb) [file 13075_2017_1418_MOESM1_ESM.docx]

**Table S1** Primer sequences used for the amplification of IL-1β 3’UTR

| miRNA | primer | sequence（5’→3’） |
| --- | --- | --- |
| miR-30c-1 | 30c wt-F | CTAGATAAGAAACCCTCTGTCATTCGCTCCCACATTCTGATGAGCAACT |
|  | 30c wt-R | CTAGAGTTGCTCATCAGAATGTGGGAGCGAATGACAGAGGGTTTCTTAT |
|  | 30c mut-F | CTAGATAAGAAACCCTCTGTCATTCGCCCTCCAATTCTGATGAGCAACT |
|  | 30c mut-R | CTAGAGTTGCTCATCAGAATTGGAGGGCGAATGACAGAGGGTTTCTTAT |
| miR-488 | 488 wt-F | CTAGAGCCAGGACAGTCAGCTCTCTCCTTTCAGGGCCAATCCCCAGCCT |
|  | 488 wt-R | CTAGAGGCTGGGGATTGGCCCTGAAAGGAGAGAGCTGACTGTCCTGGCT |
|  | 488 mut-F | CTAGAGCCAGGACAGTCAGCTCTCTCACTTCGTGGCCAATCCCCAGCCT |
|  | 488 mut-R | CTAGAGGCTGGGGATTGGCCACGAAGTGAGAGAGCTGACTGTCCTGGCT |
| miR-550a | 550 wt-F | CTAGATTTAAATCAAGTCCTTTAATTAAGACTGAAAATATATAAGCTCT |
|  | 550 wt-R | CTAGAGAGCTTATATATTTTCAGTCTTAATTAAAGGACTTGATTTAAAT |
|  | 550 mut-F | CTAGATTTAAATCAAGTCCTTTAATTAACAGGTAAAATATATAAGCTCT |
|  | 550 mut-R | CTAGAGAGCTTATATATTTTACCTGTTAATTAAAGGACTTGATTTAAAT |
| miR-663a | 663 wt-F | CTAGACCTCTCCTACTCACTTAAAGCCCGCCTGACAGAAACCACGGCCT |
|  | 663 wt-R | CTAGAGGCCGTGGTTTCTGTCAGGCGGGCTTTAAGTGAGTAGGAGAGGT |
|  | 663 mut-F | CTAGACCTCTCCTACTCACTTAAAGCCGTCCGCACAGAAACCACGGCCT |
|  | 663 mut-R | CTAGAGGCCGTGGTTTCTGTGCGGACGGCTTTAAGTGAGTAGGAGAGGT |
| miR-920 | 920 wt-F | CTAGAAAGAAACCCTCTGTCATTCGCTCCCACATTCTGATGAGCAACCT |
|  | 920 wt-R | CTAGAGGTTGCTCATCAGAATGTGGGAGCGAATGACAGAGGGTTTCTTT |
|  | 920 mut-F | CTAGAAAGAAACCCTCTGTCATTCGCTCACCACTTCTGATGAGCAACCT |
|  | 920 mut-R | CTAGAGGTTGCTCATCAGAAGTGGTGAGCGAATGACAGAGGGTTTCTTT |

The underlined nucleotides are the corresponding miRNA binding [sites](http://cn.bing.com/dict/search?q=sites&FORM=BDVSP6&mkt=zh-cn) with IL-1β 3’UTR. *wt:* wild type; *mut*: mutant type
