# Supplementary figures and images for: MicroRNA-488 and -920 regulate the production of proinflammatory cytokines in acute gouty arthritis
Source: Arthritis Res Ther. 2017 Sep 15;19:203. doi: 10.1186/s13075-017-1418-6 (PMC5602958; doi:10.1186/s13075-017-1418-6)

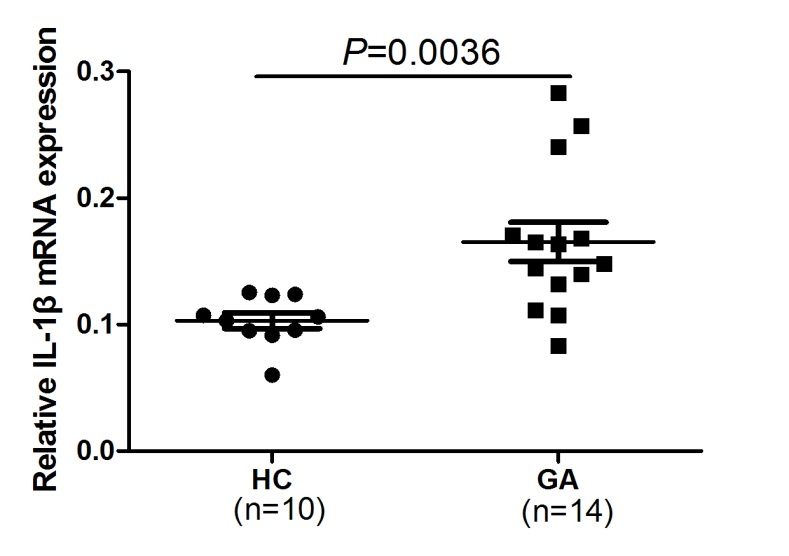

Supplement: Supplementary file 2 — IL-1β gene expression in the peripheral white blood cells of patients with acute gouty arthritis (GA, n = 14) and of healthy control subjects (HC, n = 10). IL-1β mRNA expression was detected by quantitative real-time PCR. Each bar shows the mean ± SEM. (DOCX 48 kb) [file 13075_2017_1418_MOESM2_ESM.docx]

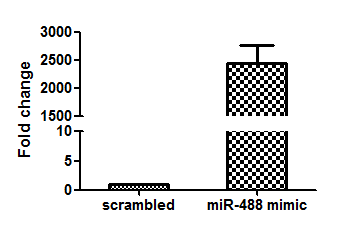

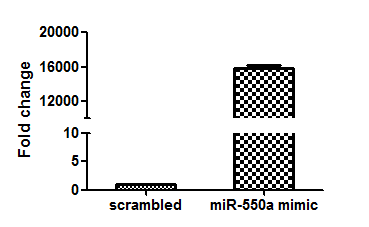

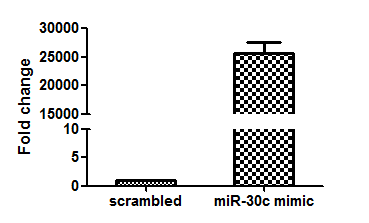


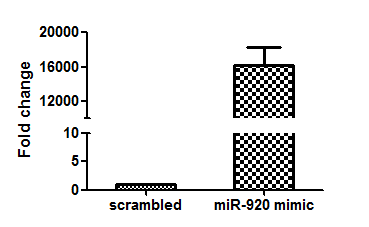

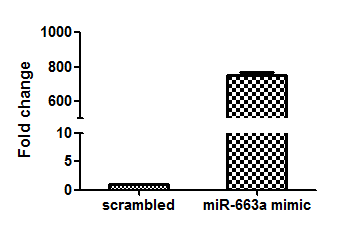

Supplement: Supplementary file 3 — THP-1 cells were transfected with 50 nM of miRNA mimics or negative control (scrambled) by using Lipofectamine RNAiMAX reagent. Expression of miRNAs was detected by quantitative real-time PCR. Each bar shows the mean ± SEM. (DOCX 31 kb) [file 13075_2017_1418_MOESM3_ESM.docx]
